# Parents’ experiences of their child’s best interests during a hospital stay in Australia

**DOI:** 10.1177/13674935241243101

**Published:** 2024-04-03

**Authors:** Angela Afua Quaye, Mandie Foster, Lisa Whitehead, Inger Kristensson Hallström

**Affiliations:** 1 Department of Health Sciences, Faculty of Medicine, 5193 Lund University, Lund, Sweden; 2 School of Clinical Sciences, 1410 Auckland University of Technology, Auckland, New Zealand; 3 School of Nursing and Midwifery, 2498 Edith Cowan University, Perth, WA, Australia; 4 Centre for Postgraduate Nursing Studies, University of Otago, Christchurch, New Zealand

**Keywords:** Child’s best interest, child-centred care, hospitalised, parents

## Abstract

Determining the child’s best interests in a hospital setting will ideally involve the combined views of children, parents, and healthcare professionals. However, few studies have explored parents’ experiences of their child’s best interests when they engage with the healthcare system. Therefore, this study aimed to explore parents’ experiences of their child’s best interests during hospitalisation. A descriptive qualitative inductive design using face-to-face parent–child combined interviews, analysed by latent content analysis, was used. Sixteen parents recruited from a tertiary hospital in Western Australia were interviewed. Collaboration, development of trustworthy relationships, and effective communication were essential in shaping parents’ experiences of their child’s best interests during hospitalisation.

## Introduction

It has been stipulated in the United Nations Convention on the Rights of the Child (UNCRC) that the child’s best interests shall be a primary consideration in all matters concerning the child (UNCRC, 1989). The child’s best interests can be elucidated from the child’s perspective and a child perspective (Quaye et al., 2021; Sommer et al., 2010). The former represents children’s understanding and experiences, highlighted in their own self-reported narratives (Foster et al., 2022; Sommer et al., 2010; Stålberg et al., 2016). The latter is defined as adults’ understanding of children’s experiences (Sommer et al., 2010).

Delivery of pediatric care worldwide has been shaped by a family-centred care (FCC) approach where an entire family unit is a care recipient (Shields et al., 2012). However, concerns have been raised over effectiveness of FCC and that it may overshadow the child’s perspective (Uniacke et al., 2018). A child-centred care (CCC) approach prioritises needs of children in a family context and recognises children as active recipients of care (Coyne et al., 2016; Foster and Shields, 2020). A CCC approach reinforces the child’s best interests and is beginning to permeate day-to-day healthcare delivery within pediatric clinical practice in Western settings (Coyne, 2014; Ford et al., 2018; Foster et al., 2019; Toly, 2017).

The bioecological model provides insight into how various factors (micro, meso, exo, and macro) in a child’s environment influence the child’s psychosocial and emotional development (Bronfenbrenner, 2004). The microsystem includes the child’s immediate environment and is the most influential system. Mesosystem includes interactions between the different components of the microsystem, whereas exosystem includes broader societal factors such as government policies and media (Bronfenbrenner, 1979). The macrosystem consists of cultural values, beliefs, and legislation as evident in the UNCRC (UNCRC, 1989). Lastly, chrono system refers to changes occurring over the child’s lifetime (Bronfenbrenner, 2004).

Hospitalisation for in-patient care is recognised globally, as stressful, and sometimes a life-changing event (Ari et al., 2019; Claridge et al., 2020; Nassery and Landgren, 2019). During hospitalisation, the hospital environment becomes part of that child’s microsystem where interactions among child, parents, and healthcare professionals (mesosystem) become of paramount importance to ensure the child’s best interests are well represented (Coker, 2018; Ford et al., 2018). In addition, the broader societal (exosystem) and cultural context (macrosystem) need to be taken into consideration to provide a holistic developmentally appropriate approach that is aligned to the child’s best interests (Logan, 2012).

Patient experiences are a valuable source of evidence to inform planning, delivery, and evaluation of healthcare services (Lygre et al., 2020). Determining the child’s best interests in a hospital setting will ideally involve combined views of children, parents, and healthcare professionals (Quaye et al., 2021). Few studies have looked at the best interests from the viewpoint of children, parents, and healthcare professionals (De Vries et al., 2013). More research is needed that explicitly explores parents’ experiences of their child’s best interests during a hospital stay. Highlighting these experiences may provide valuable information to inform pediatric practice, through facilitation of co-production of personalised care, and with a CCC approach and UNCRC principles (Aarthun et al., 2019; Carter et al., 2014; Dickinson et al., 2014; UNCRC, 1989).

## Aim

The aim was to explore parents’ experiences of their child´s best interests during hospitalisation.

## Methods

### Design

A descriptive qualitative inductive design using face-to-face parent–child combined interviews (Carter et al., 2014; Elliott and Timulak, 2005; Nisha and Michelle, 2017) was used to guide this study. Content analysis was chosen in preference to thematic analysis. Whilst thematic analysis focusses on patterns within the data (Braun and Clarke, 2006), content analysis enabled a focus on the subject, context, and emphasised variation (variation refers to the similarities within and differences between parts of the text), enabling the analysis to achieve a condensed description of the phenomenon under study (Graneheim et al., 2017; Lindgren et al., 2020). This article presents parents’ experiences; data on the children’s experiences have been previously reported (Foster et al., 2022).

### Study population and recruitment

Parents were recruited by means of convenience sampling from a tertiary hospital in Western Australia, which provides services to over 3000 children annually. One week prior to the child´s planned admission, parents were sent invitation letters by the second author with information about the study. Those who presented as an acute admission were recruited. Inclusion criteria were that parents needed to have a basic command of English language and have provided informed voluntary signed consent. Data collection took place over 7 months from June to December 2019. Interviews were conducted between 12 and 72 h post-admission and prior to discharge.

### Data collection

The parent–child interviews were conducted within the hospital premises at a time most convenient for parents, their child, and healthcare professionals. An interview guide with open-ended questions formulated from the literature was used (Dahlgren et al., 2007). Topics covered included child’s best interests, child’s participation, and interaction with parents and healthcare professionals during hospitalisation. Parents were given an opportunity to listen to their recording before transcription and to advise the researcher of any changes.

### Data analysis

Data were first analysed at a latent level. All interviews were transcribed verbatim by a research assistant and verified by the second author. The first and second authors listened to and read through each transcript to become further immersed in the data. Thereafter, the first author proceeded to identify meaning units of relevance to this study with a focus on data relating to the parents. Meaning units were then condensed to reduce text size whilst maintaining the core meaning. Condensed meaning units were then labelled with codes which were close to the original text and at low levels of abstraction and interpretation. Constant comparison of codes was conducted by the first and second authors, carefully examining similarities and differences in the data. According to Lindgren et al. (2020), data deemed rich enables abstraction of codes directly into sub-themes. Sub-themes were abstracted and pooled to form themes, independently by the first author. Discussions were held between the first and second authors multiple times, to compare, contrast, and refine sub-themes, themes, and main theme as evident in Table 1. Further discussions on the whole analytical process were held with the third and fourth authors until a consensus was reached. This analytical process was iterative, involving back-and-forth examination of meaning units and interview transcripts, in line with the study aim.

**Table 1.** An example of inductive content analysis of parents’ experiences of their child’s best interests during hospitalisation.

Meaning units	Condensed meaning units	Codes	Sub-theme	Theme
I think it’s when they can come in and you are complete strangers but look at the bigger picture and look at trying as quickly and as fast as possible to gauge a bit of info about your child and their personality type and then really work that care around that type of personality to keep their best interests at the forefront	Healthcare professionals can look at the bigger picture despite being strangers with children, and trying as quickly as possible, to gauge information about the child and their personality and work the care around the child’s personality to keep their best interest at the fore front	Knowing my child	Seeing my child beyond current illness	Tailoring care to reflect my child’s needs
Maybe come back in, in you know an hour’s time and try something again, you know, maybe just back off a little bit, leave her alone, give her some time	Mother’s opinion is for healthcare professionals to give child some time by backing off a little and leaving her alone	Giving my child space		

### Ethical approval

The children’s hospital and Edith Cowan University Ethics Committee approved this study (Number: 21943). Permission was obtained from parents for the interviews to be recorded. Parents were reminded that information they shared would be treated confidentially and that they had the right to cease participation in the study without any impact on care of their child. Pseudonyms have been used.

## Findings

The second author reached out to parents of 41 children. Of this, 16 parents representing 15 families (all European ethnicity) of children (8 girls and 7 boys) aged 2 to 14 years agreed to participate, with data saturation being reached at the 16^th^ interview. In the children’s interviews reported in a previous study (Foster et al., 2022), nine children were interviewed, and they were in the age range of 5 to 15 years. Most admissions were acute (80%), and reason for admissions included medical, surgical, and/or complications associated with a chronic illness. Reasons for non-participation included children being discharged prior to scheduled interviews or declining to participate, with interviews ranging in length from 16 to 36 min.

Analysis yielded 50 codes, 10 sub-themes, 3 themes, and 1 main theme (Table 2). The main theme ‘A balancing act of collaborating and developing trustworthy relationships through effective communication during care situations’ included three themes: (i) Supporting my child to attain increased autonomy, (ii) Tailoring care to reflect my child’s needs, and (iii) Encountering ups and downs. Direct quotes have been provided to further support themes and subthemes.

**Table 2.** Summary of the themes generated through inductive content analysis of parents’ experiences of their child’s best interests during hospitalisation.

Sub-themes	Themes	Main theme
Respecting my child’s integrity	Supporting my child to attain increased autonomy	A balancing act of collaborating and developing trustworthy relationships through effective communication during care situations
Age-appropriate interactions with my child		
Creating opportunities for my child’s active participation		
Open transparent communication		
Shared responsibilities	Tailoring care to reflect my child’s needs	
Seeing my child beyond current illness		
Advocating on my child’s behalf		
Living outside our comfort zone	Encountering ups and downs	
Positive care encounters		
Short comings in care received		

## A balancing act of collaborating and developing trustworthy relationships through effective communication during care situations

This main theme collated parents’ active and passive experiences of their child’s best interests during hospitalisation. The former refers to parents’ actions and inputs to facilitate care in their child’s best interests. The latter allude to their observations of actions of healthcare professionals, as well as of their children. Parents found themselves in situations requiring a balancing act of collaborating with their child, and healthcare professionals, to achieve intended outcomes in their child’s best interests. Navigating their new environment and healthcare system, parents depended on developing and maintaining effective communication. Effective communication was described as open and transparent communication comprising tri-directional communication involving healthcare professional–parent, healthcare professional–child, and parent–child communication. Effective communication enhanced development of relationships between the triad, described as trustworthy.

## Supporting my child to attain increased autonomy

This implied healthcare professionals interacted with the child in an age-appropriate manner that showed respect for the child’s integrity. Additionally, this meant healthcare professionals created opportunities for the child to be actively involved in their own care. This was further enhanced by occurrence of open transparent communication between child, parent, and healthcare professionals.

### Respecting my child’s integrity

For parents, respect for their child’s integrity was inferred through the attempts by healthcare professionals to seek their child’s opinions on aspects of care. This included seeking the child’s opinion when decisions needed to be made, how their child felt about an impending procedure, and how to move forward with future care situations, including preferences for route of medication delivery and food. It meant healthcare professionals sought the child’s permission prior to conducting any examinations or treatments that were invasive:

‘*They always asked for his permission, and he said yes*’

(Sally, parent of 3-year-old child).

Respecting the child’s integrity also entailed ensuring protection of their child’s privacy. For parents whose child was pubescent, the child’s preference to have a healthcare professional of the same gender as theirs or conducting routine checks without their child having to take their clothes off was appreciated. Parents were impressed and often surprised at how healthcare professionals talked to their child. Reflecting on societal changes, parents recalled their experiences as being different:

‘*It’s really good that times are changing, and we are actually talking with children as well and involving them in what’s happening to* them’

(Mary, parent of 14-year-old child).

### Age-appropriate interactions with my child

Parents shared that healthcare professionals took their child’s age and maturity into consideration, and this shaped the nature of interactions between healthcare professionals and their child. In other situations, parents felt their teenage child was ‘babied’ by healthcare professionals. Age-appropriate interactions included intentional selection of words and how healthcare professionals talked with children:

‘*When putting her needle, he* [nurse] *sat there, talked her through the whole thing. When he put it* [needle] *called it a straw, alleviated the “n” word. Sung her a song, which I thought was nice. Just spoke through a calm voice, gave a high five after*’

(Linda, parent of 6-year-old child).

Distracting children in an age-appropriate manner was described in relation to how healthcare professionals worked things out with distressed children, to gain their co-operation. Approaches included tickling, placing stickers onto reward certificates, engaging children in child-friendly play, having entertainment available such as a children’s television channel, and providing children with play activities to keep them distracted.

### Creating opportunities for my child’s participation

Supporting children to engage in their own care was a crucial element in safeguarding their best interests. Healthcare professionals encouraged the child to engage in tasks that were not too demanding for them. Minor tasks encompassed activities such as helping to take off plasters and bandages, having the child pass items from trolleys during procedures, and disposing of used items like cups or plasters. Parents saw education to self-administer medication by healthcare professionals as empowering and sustainable, especially when their child had a long-term illness and would need to continue with medication post-discharge:

‘*There’s going to be times where she needs to self-medicate. To arm her with tools to be able to do it herself and do it properly to make sure she’s breathing in the medicine, is really important*’

(Wendy, parent of 11-year-old child).

As much as parents wished for their child to participate more fully in their care, they were equally aware of factors that could limit this, such as the child’s age, situation at hand, and actions of healthcare professionals, which may limit the extent of their child’s participation:

‘*It depends on how old the child* [is]*. She’s at three and a half years old. I assume older kids is easier for them to listen to instructions and follow through. But she’s three and a half; So how to get them actively participate at this age*?’

(Mirriam, parent of 3.5-year-old child).

Nevertheless, parents still shared a wish for healthcare professionals to make ongoing efforts to engage their child in care. They believed that supporting their child’s participation in their own care created a sense of control, helped their child relax, and reduced distress and worry. Parents believed that their child’s engagement in their own care also facilitated the child’s wider engagement with healthcare professionals, adherence to planned care, and enhanced more positive experiences with healthcare delivery.

### Open transparent communication

Parents believed that healthcare professional’s use of communication styles in accordance with their child’s linguistic and developmental levels was crucial in developing open transparent communication. Parents described triadic communication during care situations as necessary and important. Open transparent communication also implied a stepwise explanation of procedures to enhance their child’s understanding of what upcoming care entailed. Sometimes parents felt that their child had no voice in matters that concerned them where healthcare professionals communicated with the parent instead of making attempts to communicate directly with their child. Having open and transparent communication allowed for sensitive issues to be explored and for the child’s concerns to be discussed:

‘*Making sure that everything is explained and it’s on their level so that they can understand, and it helps ease the stress a little bit off them*’

*(*Linda, parent of 3-year-old child).

Parents valued communication between themselves and their child and this enabled their child to share their feelings, experiences, and ask questions that they did not feel they could ask healthcare professionals. Open and transparent communication with healthcare professionals further augmented parents’ and the child’s trust in healthcare professionals. There were, however, times when parents encountered a breakdown in communication with healthcare professionals. Parents said this occurred when they were not given enough information about upcoming procedures, what would happen next, what roles were expected of them, or when parents and children did not make efforts to communicate with healthcare professionals:

‘S*ome parents and children would get that white coat syndrome and just think to shut off completely, they don’t really communicate, and it becomes very clinical*’

(Hazel, parent of 2.5-year-old child).

## Tailoring care to reflect my child’s needs

Distinguished in this theme are symbiotic efforts made by parents and healthcare professionals to provide personalised care that catered for the child’s prevailing needs, suggesting a sharing of responsibilities among the triad. Parents emphasised a need for healthcare professionals to see their child beyond the current illness, to allow healthcare professionals to know their child better from a holistic lens and implement planned care in accordance with the child’s preferences. Parents described adopting several advocacy roles orchestrated by them on their child’s behalf.

### Shared responsibilities

Shared responsibilities meant the child, parents, and healthcare professionals worked together as a team to maximise outcomes in the child’s best interests. Whilst parents attended to the daily basic needs of their child which encompassed feeding, bathing, dressing, nappy changes, and mobilisation; healthcare professionals predominantly carried out the pharmacological aspects of care. At times, parents were allowed by healthcare professionals to administer medication to their children, but this was under supervision. Frequent checks on children by healthcare professionals suggested to parents their child’s needs were at the forefront of care delivery. Shared responsibilities also inferred that in certain acute situations, parents had to give healthcare professionals space and allow them to make quick and necessary decisions during critical times, without parental interference:

‘*When we first came in it was the emergency, which I stood back and just let them do what they needed to do to get us stable*’

(Wendy, parent of 11-year-old child).

### Seeing my child beyond the current illness

Fundamental were parents’ wishes for healthcare professionals to see their child as an individual and not be defined by their current illness. Healthcare professionals needed to take a personal interest in knowing more about their child’s temperament, likes, and dislikes in addition to the medical information required. Parents described how they made efforts to help healthcare professionals better understand their child’s needs and create a good rapport with their child. In addition, parents articulated a wish for healthcare professionals to ‘*touch base*’ directly with their child, instead of using them as proxy:

‘*Look at the bigger picture and look at trying as quickly and as fast as possible to gauge a bit of info about your child and their personality type and then really work that care around that type of personality to keep their best interests at the forefront*’

(Hazel, parent of 2.5-year-old child).

Giving the child space during stressful situations implied not rushing to implement planned care when children were distressed. Parents felt it was necessary for healthcare professionals to first gauge their child’s stress levels, give the child some breathing space to process all that was going to happen, and then return after a while to continue with care as planned:

‘*Maybe come back in an hour’s time and try something again, just back off a little bit, leave her alone, give her some time*’

(Carol, parent of 6-year-old child).

### Advocating on my child’s behalf

Parents sought to help alleviate discomfort experienced by their child during hospitalisation. When parents assessed that their child’s best interests were not being honoured, they intervened by advocating on their child’s behalf. Advocacy roles included monitoring and questioning aspects of care and speaking on their child’s behalf. Parents described initiating discussions with healthcare professionals to further understand the significance and need for certain invasive procedures. Parents described the child’s best interests as pivotal when the plan of care was weighed up against alternative diagnostic tests and procedures to promote the child’s best interests and wishes. Of importance to parents was that their child’s comfort was ensured:

‘*I keep saying he'd be letting us know if somethings hurting’.*

(Leo, parent of 5-year-old child).

## Encountering ups and downs

Parents described experiencing ‘ups’ and ‘downs’. The ‘ups’ of their child’s hospital stay were defined by positive care encounters with healthcare professionals and care delivery. They met healthcare professionals who maintained a level of professionalism whilst interacting in an age-appropriate manner with their child. ‘Downs’ were described as short comings in care delivery, and parents wished for improvements. In navigating the ups and downs of their new environment, parents experienced living outside their usual comfort zone but described this as necessary to secure their child’s best interests.

### Living outside our comfort zone

Parents made sacrifices to support their child’s best interests. Parents and their child wanted to be as close as possible to each other especially when the child felt scared at night. This was not always possible, and parents grappled with feelings of losing control due to sleep deprivation and their child’s sleepless nights. Parents went through a roller coaster of emotions which they concealed to appear strong for their child. At times their child had to endure uncomfortable situations, for example, being held or the use of physical restraint was described as sometimes unavoidable, yet necessary and in the child’s best interests. Parents were aware that stress in the short term was for the greater good, indicating that they grasped the complexity involved in safeguarding their child’s best interests during hospitalisation:

‘*He is obviously feeling frustrated that he was stuck in hospital and that he was going through all this stuff, all because he needed medicine. But then you have to try and explain it to him that his best interests are to be in there to get his leg fixed*’

(Henry, parent of 3-year-old child).

### Positive care encounters

Overall, parents described themselves as content with the care received. Positive interactions were marked by a friendly and calm demeanour when healthcare professionals talked with their child. Parents appreciated how health care professionals interacted with their children. Healthcare professionals were described as welcoming, accommodating, considerate, helpful, putting in their best efforts, and having exceeded the parents’ expectations:

‘*I think they* [healthcare professional] *do an amazing job*’

(Ava, parent of 14-year-old child).

Availability of a playroom was described as instrumental in supporting their child’s best interests. Parents shared that their children felt calm and more relaxed in the playroom:

‘*Once he got up here* [playroom] a*nd he could see the toys he relaxed a bit more. Down there is a different story...Once he saw the toys in this colourful room the fish kind of calmed him down*’

(Amelia, parent of 3-year-old child).

### Shortcomings in care received

Parents grappled with variation between healthcare professionals, in relation to how their child’s best interests were brought into focus. In some situations, detailed explanations were given to children and their parents, whilst other times the information provided was not clear. Parents experienced that when healthcare professionals shared different views amongst each other, some stepped up to advocate on their behalf, for example, for less invasive procedures to be undertaken:

‘*There are physical symptoms, but she's not being cared for. You know, it hasn't been addressed. She hasn't felt looked after’.*

(Mia, parent of 5-year-old child).

Doctors were described as more distant than nurses. Long wait times associated with admission, consultation, receiving medication, undergoing examination, and procedures, led to parents feeling agitated and exhausted. Some healthcare professionals were described as task orientated and parents did not feel that their child’s best interests were safeguarded:

‘*This is my job. I’ve got a job to do. I’m going to do it without any regard for the child herself with no care for A she’s just a job*’

(Carol, parent of 6-year-old child).

## Discussion

An inductive analysis of interviews conducted with parents of hospitalised children was undertaken to explore parents’ experiences of their child’s best interests during hospitalisation. The discussion below focuses on the main components in the main theme: A balancing act of collaborating and developing trustworthy relationships through effective communication during care situations. Collaboration was recognised by parents in this study as fundamental to tailoring care to meet their child’s individual needs, a finding similarly reported in earlier research (Sundal and Vatne (2020). The process of collaboration calls for mutual negotiations (Quaye et al., 2021) of distinct roles and responsibilities to be operationalised by parents and healthcare professionals.

The process of collaboration has been described as evolving overtime from a professionally dominated encounter to a collaborative one (Swallow et al., 2013). However, healthcare professionals may be reluctant to take into consideration parental and child knowledge and expertise because of an implied shift in the balance of power (Smith et al., 2015; Swallow et al., 2013). The value of parent–healthcare professional collaborations are further reflected in the UNCRC stipulations which require parents and state parties to prioritise the child’s best interests in all matters concerning the child (UNCRC, 1989). However, the primacy of the child as an *active* agent in alliance between parent–healthcare professional collaboration must not be lost (Bronfenbrenner, 2004; Coyne et al., 2016). To safeguard the child’s best interests, collaboration requires healthcare professionals not only to draw on their professional expertise but also acknowledge and work alongside children and parents as active partners (Coyne, 2016; Oulton et al., 2020; Quaye et al., 2021).

Parents in this study wanted healthcare professionals to develop interpersonal relationships with their child and expressed wishes for healthcare professionals to look at the ‘bigger picture’ during interactions. Respecting the child’s integrity, getting to know the child at a more personal level-beyond the medical context, and giving the child space were seen as fundamental to promoting the child’s best interests. These are consistent with delivery of care within the CCC approach (Coyne et al., 2016) and are supported by Bronfenbrenner’s bioecological model in relation to the importance of understanding personal characteristics and capacities of the child as an individual (Bronfenbrenner, 2004; Rosa and Tudge, 2013). Healthcare professionals may incorporate these insights to collaborate with parents and children to help sustain a healthy bioecological system for the child during hospitalisation (Ford et al., 2018).

Developing trustworthy relationships between parents and healthcare professionals may have a positive impact on children’s experiences with healthcare (Boelsma et al., 2021; Sharkey et al., 2016). This finding is further supported by the mesosystem of the bioecological model where despite the child not being directly involved in interactions between parents and healthcare professionals, they are directly affected by these interactions (Bronfenbernner, 2004). Yet, the nature and quality of relationships developed between parents and healthcare professionals may be influenced by length of hospital stay, busy schedules and workloads of healthcare professionals, levels of parental or child stress, continuity of care provision, and severity of illness (Coyne, 2008; Mǎrginean et al., 2017). It is crucial that healthcare professionals strive to create a rapport with children and parents from their first encounter and consistently build a rapport in subsequent encounters (Sharkey et al., 2014).

The capacity to collaborate and establish and/develop trustworthy relationships in this study, hinged on effective communication. Effective communication has been discussed as fundamental to enhance patients’ health literacy (Agency for Healthcare Research and Quality, 2020; Brach et al., 2017), and it provides healthcare professionals deeper insight into patients’ symptoms, perspectives, and preferences (Street, 2016). Effective communication for parents in this study also encompassed the provision of age-appropriate information to children.

Parents of children with and without communication difficulties expressed a need for adequate communication with healthcare professionals. The experience of unmet communication needs among parents with hospitalised children is corroborated in previous research. Hemsley et al. (2013) reported that parents of children with communication difficulties felt that their child was vulnerable and more likely to be ignored during interactions with healthcare professionals. Further, parents have highlighted more generally inadequacies in the provision of, and access to, information for their children (Bray et al., 2019). Parents in this study felt that effective communication was impacted by an imbalance in power that stymied communication. These findings bring into focus the persistence of traditional imbalance of power between parents and healthcare professionals (Reeder et al., 2021) and are comparable to Carlsson et al. (2016) who reported feelings of inferiority among parents that hindered them from voicing their concerns to healthcare professionals. In accordance with UNCRC, it is an obligation for pediatric healthcare institutions to make information accessible, understandable, and that children and their parents can utilise information for the benefit of the child’s best interest (Brach et al., 2017; Foster et al., 2023).

## Limitations

Several limitations are reflected upon below. That most of the interviews were short, on average 26 min, may have narrowed scope of the data. With regards sample size, in qualitative research, an adequate sample size should be large enough to capture various experiences, whilst small enough to allow a deeper analysis leading to new and deeper understanding of experiences (Sandelowski, 1995). Therefore, a sample size of 16 participants can be considered adequate (Hennink and Kaiser, 2022).

Additionally, most admissions were acute (80%) and parental levels of stress may have influenced the depth, quality, and length of the interviews. The nature of the study did not enable the researcher to spend time with parents and build a rapport prior to conducting the interview and this may have influenced the extent to which parents freely shared their experiences. In addition, parents of children living with a disability are not fully represented in this study, and only two fathers were interviewed, hence findings may not be transferable to other settings. Despite these limitations, the processes undertaken by the authors were rigorous and followed an ethical approach to research with parents of hospitalised children.

## Implications for practice

Providers of pediatric healthcare are encouraged to engage in discussions on the roles and responsibilities of parents with their children and acknowledge their expertise to enhance collaboration. Healthcare professionals may need to continue establishing effective means of communication tailored to meet the child’s developmental and linguistic milestones. Establishing a good rapport by means of creating a friendly atmosphere, engaging in age-appropriate interactions, and building trustworthy relationships with children and their parents amidst time constraints is crucial. Planning and delivery of pediatric healthcare needs to be approached with a holistic view of children, where children are seen as individuals with emotional, physical, psychological, and social needs that extend beyond the needs related directly to the illness.

## Conclusion

This study demonstrated that collaboration, development of trustworthy relationships, and effective communication are integral aspects of healthcare delivery and essential in shaping parents’ positive experiences of their child’s best interests during hospitalisation. Tailoring communication and interaction styles in accordance with the child’s age, maturity, illness severity, emotional, cognitive, and physical developmental levels are vital in the development of effective parent and child outcomes. Future research needs to focus on including parents of children with a wider age range, type of hospital visit, diagnosis, length of hospital stays, as well as healthcare professionals’ perceptions of the child’s best interests during hospitalisation.
